# Effect of weight loss, with or without exercise, on body composition and sex hormones in postmenopausal women: the SHAPE-2 trial

**DOI:** 10.1186/s13058-015-0633-9

**Published:** 2015-09-02

**Authors:** Willemijn AM. van Gemert, Albertine J. Schuit, Job van der Palen, Anne M. May, Jolein A. Iestra, Harriet Wittink, Petra H. Peeters, Evelyn M. Monninkhof

**Affiliations:** Department of Epidemiology, Julius Centre for Health Sciences and Primary Care, University Medical Centre Utrecht, P.O. Box 85500, 3508 GA Utrecht, The Netherlands; National Institute for Public Health and the Environment, Centre for Nutrition, Prevention and Health Services, P.O. Box 1, 3720 BA Bilthoven, The Netherlands; Department of Health Sciences and EMGO Institute for Health and Care Research, VU University, Van der Boechorststraat 7, 1081 BT Amsterdam, The Netherlands; Department of Epidemiology, Medisch Spectrum Twente, P.O. Box 50000, 7500 KA Enschede, The Netherlands; Department of Research Methodology, Measurement, and Data Analysis, University of Twente, P.O. Box 217, 7500 AE Enschede, The Netherlands; Lifestyle and Health Research Group, Faculty of Health Care, Utrecht University of Applied Sciences, P.O. Box 85182, 3508 AD Utrecht, The Netherlands

## Abstract

**Introduction:**

Physical inactivity and overweight are risk factors for postmenopausal breast cancer. The effect of physical activity may be partially mediated by concordant weight loss. We studied the effect on serum sex hormones, which are known to be associated with postmenopausal breast cancer risk, that is attributable to exercise by comparing randomly obtained equivalent weight loss by following a hypocaloric diet only or mainly by exercise.

**Methods:**

Overweight, insufficiently active women were randomised to a diet (*N* = 97), mainly exercise (*N* = 98) or control group (*N* = 48). The goal of both interventions was to achieve 5–6 kg of weight loss by following a calorie-restricted diet or an intensive exercise programme combined with only a small caloric restriction. Primary outcomes after 16 weeks were serum sex hormones and sex hormone-binding globulin (SHBG). Body fat and lean mass were measured by dual-energy X-ray absorptiometry.

**Results:**

Both the diet (−4.9 kg) and mainly exercise (−5.5 kg) groups achieved the target weight loss. Loss of body fat was significantly greater with exercise versus diet (difference −1.4 kg, *P* < 0.001). In the mainly exercise arm, the reduction in free testosterone was statistically significantly greater than that of the diet arm (treatment effect ratio [TER] 0.92, *P* = 0.043), and the results were suggestive of a difference for androstenedione (TER 0.90, *P* = 0.064) and SHBG (TER 1.05, *P* = 0.070). Compared with the control arm, beneficial effects were seen with both interventions, diet and mainly exercise, respectively, on oestradiol (TER 0.86, *P* = 0.025; TER 0.83, *P* = 0.007), free oestradiol (TER 0.80, *P* = 0.002; TER 0.77, *P* < 0.001), SHBG (TER 1.14; TER 1.21, both *P* < 0.001) and free testosterone (TER 0.91, *P* = 0.069; TER = 0.84, *P* = 0.001). After adjustment for changes in body fat, intervention effects attenuated or disappeared.

**Conclusions:**

Weight loss with both interventions resulted in favourable effects on serum sex hormones, which have been shown to be associated with a decrease in postmenopausal breast cancer risk. Weight loss induced mainly by exercise additionally resulted in maintenance of lean mass, greater fitness, greater fat loss and a larger effect on (some) sex hormones. The greater fat loss likely explains the observed larger effects on sex hormones.

**Trial registration:**

ClinicalTrials.gov identifier: NCT01511276. Registered on 12 January 2012.

**Electronic supplementary material:**

The online version of this article (doi:10.1186/s13058-015-0633-9) contains supplementary material, which is available to authorized users.

## Introduction

Obesity and physical inactivity are convincing risk factors for postmenopausal breast cancer, according to the World Cancer Research Fund [1]. Together, these factors contribute to approximately 15 % of breast cancer cases that develop after menopause [2–4]. More important, these are two of the few known lifestyle-related risk factors, and, therefore, exposure is modifiable. Because the prevalence of both overweight or obesity and physical inactivity in the Western world are increasing, this subject is an important one to consider in devising breast cancer prevention strategies.

One of the pathways by which these lifestyle factors may influence postmenopausal breast cancer risk is through effects on serum sex hormones (i.e., oestrogens, androgens and the protein sex hormone-binding globulin [SHBG]) [5]. Observational, prospective studies have consistently shown a higher breast cancer risk of up to twofold in postmenopausal women with endogenous sex hormone levels in the highest versus the lowest quintile [6, 7].

In a previous randomised controlled trial, the Sex Hormones and Physical Exercise (SHAPE) study, we assessed the effect of a 1-year physical activity intervention on serum sex hormones in insufficiently active postmenopausal women. In contrast to observational studies, in which an effect of physical activity is often also observed after adjustment for differences in body mass index (BMI), we did not find an effect of exercise in the SHAPE study. However, in a subgroup of women who lost more than 2 % body fat, larger effects were observed with exercise compared with the control group. These results were supported by two other intervention studies [8–10]. We hypothesised then that weight or fat loss is essential for any beneficial effect of physical activity on sex hormones. But the question remained whether it was purely the weight loss that induced the beneficial effects on sex hormones or whether physical activity itself contributed additionally to the breast cancer risk–lowering effects. To answer this question, we designed the SHAPE-2 study, in which we are investigating the effects of an equivalent weight loss obtained by following a hypocaloric diet or mainly with an exercise programme [11]. We expect that adding exercise to diet–induced weight loss will result in a more favourable body composition (i.e., a decrease in body fat mass and a preservation or increase of lean body mass) and thereby lead to larger decreases in serum sex hormone levels compared with equivalent diet-induced weight loss. Furthermore, we hypothesise that exercise might have an effect on serum hormone levels, independent of the body fat pathway (e.g., via insulin and glucose). This hypothesis is also based on results from observational studies in which adjustments for weight still show beneficial effects of physical activity on future breast cancer risk.

## Methods

### Design and study population

The SHAPE-2 study is a three-armed, randomised controlled trial in which postmenopausal women are allocated to a diet-induced weight loss intervention, to a combined diet- and exercise-induced weight loss intervention or to a control group. The study ran from February 2012 until June 2013 in eight municipalities in the Netherlands. The study was approved by the ethics committee of the University Medical Centre Utrecht. Written informed consent was obtained from all participants. Details of the study design were reported previously [11].

Women, aged 50–69 years were recruited via mass mailings and media publicity. Women who responded were contacted by telephone by a study nurse to assess their eligibility. Eligibility was checked again during a screening visit at which weight and height were measured. Level of physical activity was further evaluated during this screening visit by focusing on sports, means of transportation and leisure time activities.

Women were eligible if they were postmenopausal (>12 months since last menses), overweight or obese (BMI 25–35 kg/m^2^) and insufficiently active (<2 h/wk of ≥4 metabolic equivalents [MET] activity) (Table 1). The main exclusion criteria were smoking, diabetes, use of exogenous (sex) hormones or ever diagnosed with breast cancer (Table 1).

**Table 1:** SHAPE-2 study inclusion and exclusion criteria

Inclusion criteria	Exclusion criteria
Female	Presently using sex hormones
Age 50–69 yr	Use of β-blockers or oral corticosteroids
Postmenopausal (>12 mo since last menses)	Smoking
Body mass index (BMI) 25–35 kg/m^2^	Alcohol or drug abuse
Insufficiently active (<2 h/wk of at least moderately intensive activities (≥4 MET))	Diagnosed breast cancer (present or history)
Willing to be randomly assigned to one of the three study arms	Diagnosed with other cancer (present or <5 yr of history), except non-melanoma skin cancer
Informed consent for all screening and study activities	Diabetes mellitus or other (unstable) endocrine-related diseases
	Any disorder that might impede participation in the exercise programme
	Following, or intention to follow, a structured weight loss programme elsewhere
	Investigator’s opinion (i.e., successful fulfilling of the programme highly unlikely)

*Source:* van Gemert et al. [11]

*Abbreviations: MET* metabolic equivalents, *SHAPE* Sex Hormones and Physical Exercise study

### Run-in period, standardised diet and randomisation

All women started with a 4–6-week run-in period during which a tailor-made, standardised diet was prescribed to maintain stable weight and to achieve a comparable diet composition among all study participants (50–60 % carbohydrates, 15–20 % proteins, 20–35 % fat, minimum 25 g of fibre, maximum of one alcoholic consumption/day) [12].

After baseline measurements, women were randomised by using a computer programme, stratified for municipality to a diet-induced weight loss group (diet group; n = 97), a weight loss induced mainly by exercise group (mainly exercise group; n = 98) or a stable weight control group (control group; n = 48). The computer programme contained an automatically generated random sequence with block sizes of 5 (2:2:1 ratio of interventions to control).

### Intervention and control group procedures

Both weight loss intervention programmes, which were aimed at achieving a 5–6-kg weight loss, were delivered by physiotherapists and/or dietitians, who also closely monitored body weight by supervised weighing. When participants reached the target weight loss, or after a maximum of 14 weeks, they entered a period of weight maintenance (2–6 weeks) wherein diet was adapted to stabilise body weight.

Steps were taken to increase and monitor compliance throughout the study period. Women filled out 3-day food diaries (in all three study groups), kept an exercise log (only in the mainly exercise group), performed weekly (only in the diet group) or biweekly (only in the mainly exercise group) self-weighing and had frequent contacts with their dietitian or physiotherapist (in the diet and the mainly exercise groups).

#### Diet group

Women in the diet group were prescribed a caloric restriction of 3500 kcal/wk (or 500 kcal/day) as compared with their estimated needs and habitual intake (standardised diet during the run-in period). They were asked to maintain their habitual physical activity levels. Individual contacts with the dietitian included two individual consultations of 30 minutes and telephone consultations every other week for monitoring and motivation [13]. During these contacts, diet was assessed by dietary history and/or by checking the food diaries. The diet was adjusted when needed. In addition, five 1-h group sessions (maximum of 12 women/group) were scheduled to provide nutritional education, self-management training and behaviour change techniques.

#### Mainly exercise group

The mainly exercise group followed an intensive 4 h/wk combined endurance and strength exercise programme. These women also were prescribed a relatively small caloric intake restriction of 1750 kcal/wk (250 kcal/day) to ensure achievement of the 5–6-kg weight loss goal within 14 weeks. Because in this group the main focus was on exercise, we refer to this group as the ‘mainly exercise induced weight loss’ or ‘mainly exercise’ group for short.

The exercise programme included two 1-h group sessions of combined strength and endurance training at the physiotherapy centre and two 1-h sessions of moderate to vigorous Nordic walking per week. Nordic Walking is a form of fitness walking enhanced by the addition of walking poles. Compared to regular walking, Nordic walking makes more use of the entire body resulting in significant increases in energy expenditure.

For a 58-year-old woman, whose weight is 78 kg and height is 1.65 m, corresponding to the average SHAPE-1 [9] participant with a BMI ≥25 kg/m^2^, the prescribed four exercise sessions resulted in an average energy expenditure of approximately 2530 kcal/wk based on corrected MET values [14, 15]. The caloric restriction was 1750 kcal/wk. The total targeted weekly energy deficit from exercise and diet in the mainly exercise group was, therefore, approximately 4280 kcal/wk.

We chose a combination of aerobic exercise and strength training because strength training leads to preservation of, or even an increase in, muscle mass and bone mineral density [16, 17]. This is important in postmenopausal women to improve health and enable an active lifestyle. Furthermore, we expected that strength training would support loss of body fat by increasing the basic metabolic rate [18].

Group sessions included 20–25 minutes of endurance training, 25 minutes of strength training and 5–10-minute warm-ups and cool-downs. Heart rate monitors were worn while exercising. Training intensity was gradually increased during the study: for endurance training, from 60 % to 90 % of the heart rate reserve [HRR]), and for strength training, based on repetition maximum tests. The 1-h Nordic walking sessions were performed at 60–65 % of HRR.

Women were strongly encouraged to join supervised classes given by a Nordic walking instructor. However, for feasibility reasons, women were also allowed to perform these sessions without guidance. Furthermore, women were instructed to increase their energy expenditure in daily activities, for example by taking the bike for shopping and by climbing stairs.

Participants kept an exercise log that the physiotherapist regularly checked.

Losing substantial weight just by increasing physical activity levels is difficult [19]. Other studies have shown that compensatory mechanisms, both physically and mentally, withhold persons from losing weight [20, 21]. To ensure substantial weight loss, a dietitian-prescribed caloric restriction of 1750 kcal/wk was added to the exercise programme [20]. The targeted total average weekly deficit for the mainly exercise group was larger than that for the diet group (4280 kcal vs 3500 kcal) to compensate for the gain in muscle mass (i.e., body weight) induced by the combined endurance and strength exercise programme.

#### Control group

Women in the control group were asked to maintain a stable weight by continuing to follow the standardised diet and their habitual physical activity patterns. To keep them from starting any attempts to lose weight during the study period, women in the control group were offered an alternative exercise weight loss programme to be started after study completion.

### Outcome measurements

Outcomes were measured at baseline (i.e., before randomisation) and at 16 weeks after baseline. Body weight, height and waist and hip circumferences were measured according to standard procedures by trained study personnel [11]. Fat and lean mass were assessed by whole-body dual-energy X-ray absorptiometry (DEXA) (Lunar iDXA, Prodigy; GE Healthcare, Little Chalfont, UK). Cardiorespiratory fitness (according to peak oxygen uptake [VO_2peak_]) was measured by having the women perform a maximal cycle exercise test during which respiratory gas analysis was done using a ramp protocol. Physical activity was measured by administering the Short Questionnaire to Assess Health-Enhancing Physical Activity (SQUASH) [22] and objectively during 7 consecutive days by having the women wear a GT3X+ activity monitor (ActiGraph, Pensacola, FL, USA) [23, 24].

#### Serum sex hormone analyses

Participants were asked not to perform moderate to vigorous physical activity in the 48 h preceding the blood sampling. Serum was collected and stored at −80 °C. After trial completion, all samples were sent, frozen, to the laboratory for analyses. Multiple samples from each individual were analysed in the same batch. Oestradiol, oestrone, androstenedione and testosterone levels were determined by liquid chromatography–mass spectrometry (LC-MS) [25] in the University Hospital of South Manchester laboratory, Manchester, UK. Free fractions of oestradiol and testosterone were calculated by using the total hormone levels, SHBG and a constant for albumin [26]. SHBG was measured by using commercially available double-antibody radioimmunoassay kits (SHBG-03052001, cobas; Roche Diagnostics, Burgess Hill, UK). The assays were performed in the SHO Velp laboratory, Velp, The Netherlands. Inter- and intra-class coefficients of variation were <10 % for androgens [27], <7 % for oestrogens [28] and <2 % for SHBG. Technicians were blinded to study allocation.

Hormone values below the lower limit of detection were assigned the value of this limit (i.e., 1.4 pg/ml for oestrone [n = 16] and 86 pg/ml for testosterone [n = 24] and androstenedione [n = 1]). Six oestradiol measures outside acceptable postmenopausal values (>42 pg/ml) and accompanying oestrone levels were excluded from analyses (five at baseline and one at follow-up).

### Statistical analyses

We calculated that 104 women in both intervention groups and 45 women in the control group were required to detect a difference of at least 8 % in oestradiol levels between the two intervention groups (primary comparison; two-sided α 0.05), and a 12–20 % decrease versus control (secondary comparison; two-sided α 0.025) with 80 % power.

The primary analysis was done according to the intention-to-treat principle. Outcomes are based on complete cases [29] (i.e., both baseline and follow-up measurements). Between-group differences in outcomes, adjusted for baseline sex hormone levels, were computed by linear regression. Hormones were log-transformed; therefore, their coefficients with 95 % confidence intervals (95 % CIs) represent a treatment effect ratio (TER) that indicates how many times the level in one group is higher (TER >1) or lower (TER <1) than the reference group.

Secondary analyses were performed in (1) women who reached >2-kg weight loss in the intervention groups and stable weight (±2 kg) in the control group and (2) women who adhered to the exercise goal (i.e., for the mainly exercise group, >80 % attendance; for the diet and control groups, <60-minute increase in leisure time activities of ≥4 MET/wk, according to the SQUASH questionnaire or, if missing, ActiGraph activity monitor). IBM SPSS statistical software (version 20; IBM, Armonk, NY, USA) was used for the analyses.

## Results

Women in the intervention and control groups were comparable in baseline characteristics (Table 2). Study participants had a mean age of 60 years, a mean BMI of 29.2 kg/m^2^, a mean body fat percentage of 44 % and a mean VO_2peak_ of 21.9 ml/kg/min, indicating poor physical fitness.

**Table 2:** Baseline characteristics of the SHAPE-2 study population

		Control group	Diet group	Mainly exercise group
		(n = 48)	(n = 97)	(n = 98)
		Mean (SD)		
Age (yr)		60.0±4.9	60.5±4.6	59.5±4.9
Time since last menses (yr)		11.4±7.8	10.7±6.1	10.9±7.7
Education level,^a^ n (%)				
	Low	15 (31.3 %)	27 (27.8 %)	33 (33.6 %)
	Middle	15 (31.3 %)	27 (27.8 %)	20 (20.4 %)
	High	18 (37.5 %)	42 (43.3 %)	44 (44.9 %)
First-degree family member(s) with breast cancer, n (%)		9 (18.8 %)	23 (23.7 %)	24 (24.5 %)
Anthropometrics				
	Weight (kg)	80.9±10.0	80.0±8.6	80.4±9.0
	BMI (kg/m^2^)	29.5±2.6	29.3±2.5	29.0±2.9
	Waist circumference (cm)	99.0±8.7	97.8±7.5	97.5±8.3
	Hip circumference (cm)	110±7.7	110±6.8	109±6.7
Body composition measured by DEXA				
	Body fat percentage (%)	43.6±5.0	44.1±3.8	43.8±4.0
	Total body fat (kg)	34.2±7.4	33.9±5.7	33.9±6.2
	Lean mass (kg)	43.4±3.9	42.7±4.0	43.1±4.1
Physical fitness and activity				
	VO_2peak_, relative (ml/kg/min)	22.1±4.7	21.9±4.0	21.8±3.7
	VO_2peak_ (ml/min)	1751±363	1742±310	1749±293
	Physical activity monitor (min/day)^b^	Median (IQR)		
	Sedentary	652 (600–691)	637 (606–685)	630 (593–678)
	Light	179 (164–226)	194 (175–214)	197 (157–229)
	Moderate	35 (25–39)	35 (22–46)	33 (27–46)
	Vigorous	0.33 (0.17–0.61)	0.35 (0.17–0.53)	0.29 (0.14–0.47)
	SQUASH moderate and vigorous activity^c^ (min/wk)	270 (120–495)	184 (115–420)	248 (90–465)
Alcohol (g/day)		3.7 (0.0–11.7)	5.7 (0.0–10.0)	4.3 (0.0–10.0)
		Geometric mean (95 % CI)		
Oestradiol (pg/ml)		4.10 (3.51–4.79)	4.15 (3.67–4.70)	3.70 (3.33–4.12)
Oestrone (pg/ml)		21.0 (18.4–24.0)	20.4 (18.9–22.0)	19.3 (17.7–21.1)
Free oestradiol (pg/ml)		0.10 (0.08–0.12)	0.10 (0.08–0.11)	0.09 (0.08–0.10)
Testosterone (pg/ml)		201 (174–233)	196 (178–215)	183 (167–200)
Androstenedione (pg/ml)		593 (508–692)	561 (508–620)	556 (497–622)
Free testosterone (pg/ml)		2.78 (2.36–3.28)	2.54 (2.31–2.79)	2.41 (2.21–2.63)
SHBG (nmol/L)		45.1 (39.7–51.3)	50.1 (45.7–55.0)	48.8 (44.7–53.3)

Data on family history of breast cancer were available for 241 women (99.2 %), DEXA scan measurements for 240 women (98.8 %), VO_2peak_ for 237 women (97.5 %), alcohol intake for 226 women (93.0 %), SQUASH questionnaires for 236 (97.1 %) women and accelerometer data for 161 of 215 women (74.9 %). All hormone levels were missing for one woman, and (free) oestradiol, (free) testosterone and androstenedione were also missing for one woman. Five baseline values for (free) oestradiol and oestrone were excluded (>42 pg/ml). All other data were available for all women (N = 243)

*Abbreviations: BMI* body mass index, *CI* confidence interval, *DEXA* dual-energy X-ray absorptiometry, *IQR* interquartile range, *SD* standard deviation, *SHAPE* Sex Hormones and Physical Exercise study, *SHBG* sex hormone-binding globulin, *SQUASH* Short Questionnaire to Assess Health-Enhancing Physical Activity, *VO*_*2peak*_ peak oxygen uptake

^a^Education levels: low = primary school and technical/professional school, middle = college degree, high = university degree

^b^GT3X+ ActiGraph activity monitor measuring minutes per day of activity spent in each activity category

^c^Based on the SQUASH physical activity questionnaire, activities performed ≥4 metabolic equivalents

**Fig. 1:**
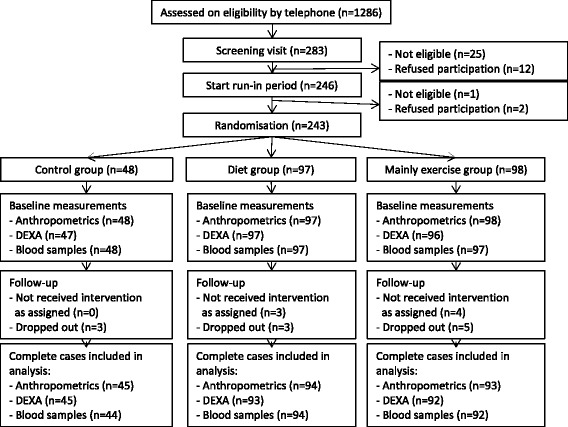
Flowchart of the inclusion, random assignment and follow-up of the Sex Hormones and Physical Exercise (SHAPE)-2 study participants. ‘Dropouts’ refers to women who withdrew from the study before the end of the study and who did not participate in follow-up measurements. ‘Not received intervention as assigned’ refers to women who also withdrew from the study prematurely, but who attended follow-up measurements. *DEXA* dual-energy X-ray absorptiometry

Of all the 243 participating women, 232 (95.5 %) completed the trial and 11 dropped out (three each in the control and diet groups and five in the mainly exercise group) (see Fig. 1). Complete data on weight, BMI, and waist and hip circumferences were available for 232 women; fat mass (in kilograms and percent) and lean mass for 230 women; VO_2peak_ for 219 women; and SQUASH data for 206 women. Blood samples of 230 women were available. The median number of group sessions attended by women in the diet group was four (out of five offered), and 70 % of women attended at least four sessions. All women attended the first individual contact appointment with the dietitian for the standardised diet prescription. Of the women in the intervention groups, 98.4 % attended the individual consultation with the dietitian for the intervention diet prescription and 91.4 % attended the individual consultation for maintenance diet prescription. All other women received dietary prescriptions by post, which were discussed by telephone. For the mainly exercise group, the median attendance rates for the group exercise sessions and Nordic walking training hours were 81 % and 88 %, respectively. Musculoskeletal injuries were reported by 9 % in the control group, 5 % in the diet group and 15 % in the mainly exercise group. No serious adverse events occurred.

### Body composition and fitness outcomes

After 16 weeks, the diet and mainly exercise groups accomplished average weight loss of −4.9 kg (−6.1 %) and −5.5 kg (−6.9 %), respectively. The control group remained weight-stable (0.06 kg [0.1 %]) (Table 3). All anthropometric factors and body fat (in kilograms and percent) showed statistically significant decreases in both intervention groups versus the control group (Table 3).

**Table 3:** Baseline and 16-week differences in body composition and fitness between study groups

		Baseline mean	16-wk mean	Change at 16 wk	Percent change at 16 wk	Treatment effect^a^ (95 % CI), intervention vs control	*P* value*	Treatment effect^a^ (95 % CI), mainly exercise vs diet	*P* value**
Body weight (kg)									
	Control	80.4	80.4	0.06	0.07				
	Diet	80.3	75.4	−4.89	−6.09	−4.95 (−5.69 to −4.21)	<0.001		
	Mainly exercise	80.4	74.9	−5.52	−6.87	−5.58 (−6.32 to −4.84)	<0.001	−0.63 (−1.23 to −0.04)	0.037
BMI (kg/m^2^)									
	Control	29.3	29.4	0.02	0.08				
	Diet	29.2	27.5	−1.78	−6.07	−1.80 (−2.06 to −1.53)	<0.001		
	Mainly exercise	29.0	27.0	−2.00	−6.88	−2.02 (−2.29 to −1.75)	<0.001	−0.22 (−0.44 to −0.01)	0.044
Waist circumference (cm)									
	Control	98.6	97.9	−0.66	−0.67				
	Diet	97.9	92.7	−5.14	−5.25	−4.54 (−5.76 to −3.33)	<0.001		
	Mainly exercise	97.5	91.6	−5.97	−6.12	−5.40 (−6.62 to 4.18)	<0.001	−0.86 (−1.84 to 0.13)	0.087
Hip circumference (cm)									
	Control	109.2	109.6	0.35	0.32				
	Diet	109.9	105.9	−3.99	−3.63	−4.31 (−5.30 to −3.32)	<0.001		
	Mainly exercise	109.1	104.8	−4.31	−3.95	−4.67 (−5.65 to −3.68)	<0.001	−0.36 (−1.15 to 0.44)	0.377
Body fat percentage (%)									
	Control	43.5	43.7	0.22	0.50				
	Diet	44.0	41.5	−2.54	−5.76	−2.82 (−3.54 to −2.11)	<0.001		
	Mainly exercise	43.9	39.8	−4.11	−9.38	−4.38 (−5.10 to −3.67)	<0.001	−1.56 (−2.14 to −0.98)	<0.001
Total body fat (kg)									
	Control	33.8	34.0	0.17	0.49				
	Diet	34.0	30.3	−3.70	−10.89	−3.87 (−4.60 to −3.14)	<0.001		
	Mainly exercise	34.0	28.8	−5.13	−15.11	−5.30 (−6.03 to −4.56)	<0.001	−1.43 (−2.02 to −0.84)	<0.001
Lean mass (kg)									
	Control	43.3	43.2	−0.10	−0.22				
	Diet	42.9	42.1	−0.78	−1.82	−0.71 (−1.14 to −0.23)	<0.001		
	Mainly exercise	43.0	43.0	−0.06	−0.14	0.02 (−0.42 to 0.46)	0.930	0.73 (0.38–1.08)	<0.001
VO_2peak_ (ml/min)									
	Control	1761	1682	−78.6	−4.46				
	Diet	1752	1707	−44.9	−2.56	32.0 (−29.9 to 93.8)	0.310		
	Mainly exercise	1766	1885	118.7	6.72	198.4 (136.6–260.1)	<0.001	166.4 (116.8–216.0)	<0.001
SQUASH moderate and vigorous activity^b^ (min/wk)									
	Control	270	300	−30.0	−11.1				
	Diet	184	170	−14.0	−7.6	−82.6 (−263.8 to 98.6)	0.370		
	Mainly exercise	248	495	247	99.6	221.7 (42.9–400.5)	0.015	304.3 (157.9–450.7)	<0.001

Baseline and follow-up measurements of complete cases (i.e. women with both baseline and follow-up measurements) are presented. Complete case data of weight, BMI and waist and hip circumferences were available for 232 women; fat mass (kg and %) and lean mass for 230 women; VO_2peak_ for 219 women; and SQUASH for 206 women

*Abbreviations: BMI* body mass index, *CI* confidence interval, *SQUASH* Short Questionnaire to Assess Health-Enhancing Physical Activity, *VO*_*2peak*_ peak oxygen uptake

*P < 0.025 was considered significant for the comparison of both intervention groups vs control

**P < 0.05 was considered significant for the comparison mainly exercise vs diet

^a^Treatment effect (95 % confidence interval) is the regression coefficient of a linear regression analysis

^b^Based on the SQUASH physical activity questionnaire, activities performed ≥4 metabolic equivalents

Compared with the diet group, the mainly exercise group showed a greater decrease in waist and hip circumferences; however, the differences were not statistically significant (respectively: −0.86 cm, 95 % CI −1.84 to 0.13; −0.36 cm, 95 % CI −1.15 to 0.44). Decreases in body fat were statistically significantly greater in the mainly exercise versus the diet group (respectively: −1.43 kg, 95 % CI −2.02 to −0.84; −1.56 %, 95 % CI −2.14 to −0.98). Lean mass was preserved in the mainly exercise group compared with control (0.02 kg, 95 % CI −0.42 to 0.46), whereas the diet group lost lean mass (−0.71, 95 % CI −1.14 to −0.23). VO_2peak_ was statistically significantly increased in women in the mainly exercise group by 198 ml/min (95 % CI 137–260) versus the control group and by 32.0 ml/min (95 % CI −29.9 to 93.8) compared with the diet group. Moderate to vigorous physical activity according to the SQUASH questionnaire also increased in the mainly exercise group by 222 min/wk (95 % CI 43–401) compared with the control arm and by 304 min/wk (95 % CI 158–451) compared with the diet group.

### Sex hormone outcomes

In our primary comparison, exercise versus diet-induced weight loss, the mainly exercise group showed larger treatment effects (Table 4). Statistically significantly larger effects were found for free testosterone (TER 0.92, 95 % CI 0.85–0.99), and borderline statistically significant effects were seen for androstenedione (TER 0.90, 95 % CI 0.80–1.01) and SHBG (TER 1.05, 95 % CI 1.00–1.12) (Table 4).

**Table 4:** Baseline and 16-week differences in serum sex hormones and treatment effects between study groups

		Baseline geometric mean	Geometric mean at 16 wk	Change at 16 wk, %	TER^a^ (95 % CI), intervention vs control	*P* value*	TER^a^ (95 % CI), mainly exercise vs diet	*P* value**
Oestradiol (pg/ml)								
	Control	3.89	4.01	3.11				
	Diet	4.20	3.62	−13.8	0.86 (0.75–0.98)	0.025		
	Mainly exercise	3.69	3.22	−12.7	0.83 (0.73–0.95)	0.007	0.97 (0.87–1.08)	0.562
Oestrone (pg/ml)								
	Control	20.1	20.4	1.51				
	Diet	20.4	20.1	−1.26	0.98 (0.88–1.08)	0.650		
	Mainly exercise	19.9	18.5	−6.67	0.92 (0.82–1.02)	0.109	0.94 (0.86–1.02)	0.154
Free oestradiol (pg/ml)								
	Control	0.09	0.10	3.23				
	Diet	0.10	0.08	−17.7	0.80 (0.70–0.92)	0.002		
	Mainly exercise	0.09	0.07	−19.1	0.77 (0.67–0.88)	<0.001	0.96 (0.85–1.07)	0.425
Testosterone (pg/ml)								
	Control	194	186	−4.07				
	Diet	197	189	−3.76	1.01 (0.92–1.10)	0.886		
	Mainly exercise	186	172	−7.63	0.96 (0.87–1.05)	0.332	0.95 (0.88–1.02)	0.166
Androstenedione (pg/ml)								
	Control	575	560	−2.60				
	Diet	562	537	−4.50	0.97 (0.85–1.12)	0.684		
	Mainly exercise	573	488	−14.7	0.87 (0.76–1.00)	0.059	0.90 (0.80–1.01)	0.064
Free testosterone (pg/ml)								
	Control	2.71	2.61	−3.90				
	Diet	2.53	2.25	−11.2	0.91 (0.83–1.01)	0.069		
	Mainly exercise	2.44	2.01	−17.7	0.84 (0.76–0.93)	0.001	0.92 (0.85–0.99)	0.043
SHBG (nmol/L)								
	Control	44.2	44.0	−0.30				
	Diet	50.7	57.1	12.6	1.14 (1.07–1.23)	<0.001		
	Mainly exercise	49.3	58.6	19.0	1.21 (1.12–1.30)	<0.001	1.05 (1.00–1.12)	0.070

Baseline and follow-up measurements of complete cases (i.e., women with both baseline and follow-up measurements) are presented. Complete case data of oestradiol were available for 223 women; oestrone for 221 women; free oestradiol for 222 women; testosterone and androstenedione for 229 women; free testosterone for 228 women; and SHBG for 230 women

*Abbreviations: CI* confidence interval, *SHBG* sex hormone-binding hormone, *TER* treatment effect ratio

*P < 0.025 was considered significant for the comparison of both intervention groups vs control

**P < 0.05 was considered significant for the comparison mainly exercise vs diet

^a^TER represents the overall intervention effect on hormone change (adjusted for baseline), estimated by linear regression analysis. Because the linear regression models were based on log-transformed hormone data, the presented treatment effect is the antilogarithm of the original estimate. Therefore, the TER is a ratio that indicates how many times the level in one group is higher (TER >1) or lower (TER <1) than a reference group. For example, TER intervention vs control of 0.9 indicates that the hormone level in the intervention group is, on average, 10 % lower than in the control group

When we compared both intervention groups with the control group, our secondary comparison, we found that all hormone levels had decreased and SHBG had increased (beneficial), except for testosterone in the diet group (Table 4). Of these changes, statistically significant effects were found for oestradiol (bound and free) and SHBG in both the diet and mainly exercise groups and for free testosterone in the mainly exercise group. Borderline statistically significant changes were found for free testosterone in the diet group versus the control group and for androstenedione in the mainly exercise group versus the control group.

After adjustment for changes in body fat percentage, the observed intervention effects on (free) oestradiol, oestrone, free testosterone and SHBG were attenuated or disappeared (Additional file 1). Only for androstenedione were the additional effects of exercise versus diet not substantially changed (TER 0.90, 95 % CI 0.79–1.01; *P* = 0.071).

In a secondary analysis of women who lost more than 2 kg (intervention groups) or remained weight-stable (control group) (n = 206), results on sex hormones and SHBG were comparable to those in the intention-to-treat analysis (Additional file 2). A per-protocol analysis of women adherent to the exercise prescription (n = 168) showed larger intervention effects on all hormones. We defined adherence as >80 % attendance at all exercise classes for the mainly exercise group. For the diet and control groups, adherence was defined as <60 min/wk increase in moderate to vigorous activities (≥4 MET), based on the SQUASH questionnaire or, if missing, on the ActiGraph activity monitor data.

The additional effect of exercise on SHBG compared with diet increased to a significant level, whereas the additional effects of androstenedione and free testosterone disappeared (Additional file 3).

## Discussion

We found that, in postmenopausal women, 6–7 % weight loss induced mainly by exercise resulted in a more favourable body composition (i.e., larger loss of body fat and preservation of lean mass), better physical fitness and larger effects on free testosterone, as well as suggestive effects for androstenedione and SHBG compared with a similar amount of weight loss induced by following a hypocaloric diet only. The exercise intervention consisted of a combination of endurance and strength training. Furthermore, both weight loss interventions resulted in significant favourable effects on oestradiol, free oestradiol, androstenedione (exercise only), free testosterone and SHBG compared with the control group. After adjustment for changes in body fat, most intervention effects were attenuated or disappeared. These findings support the hypothesis that the greater loss of body fat induced by exercise compared with diet largely mediates the effects of physical activity on sex hormones.

A modest and sustained body weight reduction of 3–5 % has been shown to result in clinically meaningful improvements in health, and the degree of weight loss is directly proportional to health benefits regarding cardiovascular outcomes [30]. Large cohort studies also suggest that modest weigh loss (5–10 %) results in breast cancer risk reductions of 25–50 % [31, 32]. Our results show that a reduction in body fat, more than a decrease in body weight in general, is an important factor in inducing changes in sex hormones. These findings contribute to the body of evidence in this field derived from the previous exercise or weight loss intervention studies in postmenopausal women [8–10, 33] and to the understanding of the underlying mechanisms connecting physical activity and decreased breast cancer risk. After menopause, fat tissue is the most important source of oestrogens because the enzyme aromatase, present in adipose tissue, converts androgens to oestrogens [34]. Furthermore, abdominal fat is associated with higher levels of insulin, inhibiting SHBG production [35, 36]. In our study, both intervention groups experienced a decrease in fat tissue, but more so in the mainly exercise group than in the diet group, despite comparable weight loss. The fat loss induced a decrease in sex steroid hormone levels and an increase in SHBG, resulting in less unbound and biologically active oestradiol and testosterone. Two randomised, low-fat dietary intervention studies demonstrated small weight losses (3–6 %), and these trials also showed significant improvements in SHBG [37, 38] and testosterone [37], but not in oestradiol.

A study comparable to ours is the Nutrition and Exercise for Women (NEW) trial, a 12-month study of postmenopausal women in the United States in which researchers are investigating the combined and individual effects of a diet and aerobic exercise intervention on sex hormones [39]. Unlike SHAPE-2, the aim of that trial was not equivalent weight loss, and SHAPE-2 also included resistance training in addition to aerobic exercise. In the NEW trial, the combined exercise and diet group (most comparable to our mainly exercise group) had the largest losses in body weight (−9.8 kg) and body fat (−6.4 %), whereas diet alone resulted in −9.1 kg weight loss and −5.0 % fat loss [39]. In the exercise-alone group, these figures were −2.8 kg and −2.1 %, respectively. All three intervention groups showed significant effects on sex hormones compared with the control group. The exercise-only group showed the smallest effects. The diet and the diet with exercise group showed larger changes in sex hormones. Except for the changes in androgens, changes in oestrogens and SHBG in the NEW diet intervention groups were larger than in our study (5–10 % difference on average). For example, free oestradiol was reduced 21 % in the NEW diet group versus 18 % in our diet group and by 26 % in the NEW diet with exercise group versus 19 % in our mainly exercise group. These findings are in agreement with their larger losses in fat mass compared with the SHAPE-2 data, indicating a dose–response relationship for oestrogens. However, in the NEW trial, none of the differences between the diet group and diet plus exercise group reached statistical significance.

We found additional effects of exercise compared with diet alone on androgens and SHBG. For oestrogens, we observed small differences (3–4 %) that were not statistically significant. Our study was powered to detect a difference of 8 % as a minimum, which could explain the non-significance of these findings. The NEW trial researchers concluded that greater weight loss produced stronger effects on oestrogens and SHBG. The SHAPE-2 trial adds to their conclusion that, more specifically, fat loss produces stronger effects on sex hormones, including androgens.

Losing weight mainly by exercise instead of by diet alone resulted in a larger loss of fat, the target tissue for relevant biomarkers, and preservation of lean body mass, which is important for prevention of other chronic diseases. It is known that sarcopenia, characterised by a loss of lean mass, often affects elderly persons and is responsible for high morbidity and mortality [40].

Exercise can be roughly divided into two types: endurance and strength training. Endurance training is most likely to result in weight loss [41]. However, especially strength training is associated with a more favourable body composition regarding total fat and muscle mass [17, 41]. Therefore, to achieve weight reduction, we recommend a combination of diet with exercise, supporting current recommendations on lifestyle behaviour change to reduce obesity [42, 43].

To our knowledge, no previous researchers have reported on effects of strength training alone on sex hormones in postmenopausal women. In our study, all women in the mainly exercise group engaged in both types of exercise; thus, we are not able to disentangle separate effects of endurance and strength training.

Most observational studies show an independent effect of physical activity, after adjusting for body weight, on breast cancer risk [1, 44, 45]. Some also have found an independent effect on serum sex hormones [46, 47]. This may reflect residual confounding because adjustment for weight or BMI does not fully cover the adjustment for fat, the most relevant tissue. Another explanation is that exercise affects other breast cancer risk–related mechanisms which are not (fully) fat-dependent, such as insulin sensitivity or the immune system and inflammation [5].

The direct impact of our study results on breast cancer risk remains speculative. We used aromatase inhibitor and BMI studies to estimate the clinical impact. Aromatase inhibitors reduce oestradiol by 83–89 % in patients with breast cancer [48–50], whereas researchers in two randomised trials in healthy high-risk women observed a 53–65 % breast cancer risk reduction associated with these drugs during 5 years of follow-up [51, 52]. Extrapolating these data to our study, wherein we observed a 13 % decrease in oestradiol, would reveal an 8–10 % reduction in breast cancer risk. The observed two-unit reduction in BMI as a starting point would reduce breast cancer risk by approximately 5 % because every five-unit gain in BMI shows a relative risk of 1.13 [1]. Although these different estimation methods and cohort studies [31, 32] indicate a 5–10 % risk reduction, the direct and long-term impacts of weight loss on breast cancer risk are still unclear, leaving a challenge for future research.

Our study has several strengths. First, we used a strong design with the unique aim of reaching comparable weight loss between the two intervention groups, which was largely accomplished in both groups. In addition, our study design incorporated a run-in period during which all women were prescribed a standardised diet. Therefore, food components that might potentially influence sex hormones, such as alcohol and dietary fibre, are unlikely to have affected the results. Another strength is the high adherence to the study protocol in all three groups. Adherence of the control group is often challenging in lifestyle-related trials [53]; therefore, we offered an alternative weight loss programme after trial completion. Finally, we used the LC-MS method, which is the reference standard because it is a highly sensitive technique to measure hormone levels that is less prone to cross-reactions [54, 55].

There are also some limitations which we need to acknowledge. Despite the fact that both intervention groups achieved the weight loss target, there was a difference of 0.6 kg in favour of the mainly exercise group. Although this is a clinically small difference, it may have affected the outcomes related to the exercise–diet comparison slightly. However, the difference in fat loss we observed to be most influential on sex hormones was much larger between the two groups. Furthermore, as weight loss represents mainly fat loss, additional adjustment for weight change has no added value.

## Conclusions

We found that a modest reduction in body weight (6–7 %) either by following a hypocaloric diet or mainly by exercise led to beneficial effects on sex hormones and SHBG. Moreover, this amount of weight loss induced mainly by exercise led to a more favourable body composition (less fat and preservation of lean mass) and free testosterone, androstenedione (lower) and SHBG (higher). Body fat largely mediated the effects of exercise on these hormones, suggesting that fat loss in particular is most important in influencing sex hormone levels which are associated with postmenopausal breast cancer risk.

## Additional files

Additional file 1:
**Treatment effects on sex hormones, adjusted for changes in fat percentage.** Analyses done to get insight into whether effects on sex hormones are mediated by changes in body fat. (PDF 40.8kb) Additional file 2:
**Effects on sex hormones in women who lost >2 kg of body weight (intervention groups) or remained weight-stable (±2 kg; control group).** Secondary analyses of women who lost body weight or remained weight-stable. (PDF 31.5 kb) Additional file 3:
**Effects on sex hormones in women who were adherent to the prescribed physical activity.** Secondary analyses of women adherent to the physical activity prescription. (PDF 60.4 kb)

## Abbreviations

BMI: Body mass index
CI: Confidence interval
DEXA: Dual-energy X-ray absorptiometry
HRR: Heart rate reserve
LC-MS: Liquid chromatography–mass spectrometry
MET: Metabolic equivalents
NEW: Nutrition and Exercise for Women trial
SHAPE: Sex Hormones and Physical Exercise study
SHBG: Sex hormone-binding globulin
SQUASH: Short Questionnaire to Assess Health-Enhancing Physical Activity
TER: Treatment effect ratio
VO_2 peak_: Peak oxygen uptake
