# Supplementary material for: Effect of weight loss, with or without exercise, on body composition and sex hormones in postmenopausal women: the SHAPE-2 trial
Source: Breast Cancer Res. 2015 Sep 2;17(1):120. doi: 10.1186/s13058-015-0633-9 (PMC4557857; doi:10.1186/s13058-015-0633-9)
Supplement: Additional file 1: — Treatment effects on sex hormones, adjusted for changes in fat percentage. Analyses done to get insight into whether effects on sex hormones are mediated by changes in body fat. (PDF 40.8kb) [file 13058_2015_633_MOESM1_ESM.pdf]

Additional File 1. Treatment effects on sex hormones, adjusted for changes in fat percentage

	TER* (95% CI): Intervention vs. Control	*P*-value[a]	TER* (95% CI): Mainly e~~E~~xercise vs. Diet	*P*-value[b]
Oestradiol (pg/mL)				
Diet	0.96 (0.83 to 1.10)	0.530		
Mainly e~~E~~xercise	0.98 (0.83 to 1.16)	0.814	1.03 (0.92 to 1.15)	0.650
Oestrone (pg/mL)				
Diet	1.04 (0.93 to 1.16)	0.500		
Mainly ~~E~~exercise	0.99 (0.87 to 1.13)	0.925	0.96 (0.88 to 0.96)	0.320
Free oestradiol (pg/mL)				
Diet	0.92 (0.79 to 1.06)	0.238		

Mainly ~~E~~exercise	0.94 (0.79 to 1.12)	0.490	1.03 (0.92 to 1.16)	0.631
Testosterone (pg/mL)				
Diet	1.01 (0.91 to 1.12)	0.810		
Mainly ~~E~~exercise	0.96 (0.85 to 1.08)	0.464	0.94 (0.87 to 1.02)	0.166
Androstenedione (pg/mL)				
Diet	1.00 (0.85 to 1.16)	0.949		
Mainly e~~E~~xercise	0.89 (0.75 to 1.06)	0.198	0.90 (0.79 to 1.01)	0.071
Free testosterone (pg/mL)				
Diet	0.96 (0.87 to 1.07)	0.511		
Mainly e~~E~~xercise	0.91 (0.81 to 1.03)	0.150	0.95 (0.87 to 1.09)	0.201
SHBG (nmol/L)				
Diet	1.07 (0.99 to 1.16)	0.069		

Mainly e~~E~~xercise	1.08 (0.99 to 1.18)	0.070	1.01 (0.95 to 1.07)	0.747

a. A *P*-value of <0.025 was considered significant for the comparison of both intervention groups versus control.

b. A *P*-value of <0.05 was considered significant for the comparison mainly exercise versus diet.

Complete case data of oestradiol were available for 223 women; oestrone for 221 women; free oestradiol for 222 women; testosterone and androstenedione for 229 women; free testosterone for 228 women; and SHBG for 230.

*TER=Treatment effect ratio (95% confidence interval), which represents the overall intervention effect on hormone change (adjusted for baseline), estimated by linear regression analysis. Because the linear regression models were based on log-transformed hormone data, the presented treatment effect is the antilogarithm of the original estimate. Therefore, the TER is a ratio that indicates how many times the level in one group is higher (TER>1) or lower (TER<1) compared with a reference group. For example, TER intervention versus control = 0.9 indicates that the hormone level in the intervention group is on average 10% lower compared with the control group.
