# Supplementary material for: Effect of weight loss, with or without exercise, on body composition and sex hormones in postmenopausal women: the SHAPE-2 trial
Source: Breast Cancer Res. 2015 Sep 2;17(1):120. doi: 10.1186/s13058-015-0633-9 (PMC4557857; doi:10.1186/s13058-015-0633-9)
Supplement: Additional file 2: — Effects on sex hormones in women who lost >2 kg of body weight (intervention groups) or remained weight-stable (±2 kg; control group). Secondary analyses of women who lost body weight or remained weight-stable. (PDF 31.5 kb) [file 13058_2015_633_MOESM2_ESM.pdf]

Additional File 2. Effects on sex hormones in women who lost >2 kg body weight (intervention groups), or remained weight stable (±2 kg, control group)

	Baseline Geometric mean	16 weeks Geometric mean	% change 16 weeks	TER* (95% CI): Intervention vs. Control	P-value#	TER* (95% CI): Mainly eExercise vs. Diet	P-value^
Estradiol (pg/ml)							
Control	3.80	4.00	5.18				
Diet	4.33	3.69	-14.6	0.85 (0.73 to 0.98)	0.030		
Mainly eExercise	3.73	3.23	-13.3	0.82 (0.71 to 0.95)	0.008	0.96 (0.86 to 1.08)	0.532
Estrone (pg/ml)							
Control	20.1	20.6	2.65				
Diet	20.6	20.3	-1.56	0.97 (0.87 to 1.07)	0.507		
Mainly eExercise	20.2	18.9	-6.20	0.91 (0.82 to 1.02)	0.095	0.95 (0.87 to 1.03)	0.192
Free estradiol (pg/ml)							
Control	0.09	0.10	4.31				
Diet	0.10	0.08	-19.4	0.79 (0.68 to 0.92)	0.002		

Mainly eExercise	0.09	0.07	-20.2	0.75 (0.65 to 0.88)	<0.001	0.95 (0.85 to 1.07)	0.432
Testosterone (pg/ml)							
Control	193	189	-2.33				
Diet	198	190	-4.17	0.99 (0.89 to 1.09)	0.780		
Mainly eExercise	186	174	-6.91	0.95 (0.86 to 1.04)	0.262	0.96 (0.89 to 1.03)	0.272
Androstenedione (pg/ml)							
Control	582	554	-4.67				
Diet	567	538	-5.27	0.98 (0.85 to 1.14)	0.834		
Mainly eExercise	576	505	-12.3	0.92 (0.79 to 1.06)	0.249	0.93 (0.83 to 1.04)	0.228
Free testosterone (pg/ml)							
Control	2.78	2.67	-3.70				
Diet	2.58	2.24	-13.3	0.89 (0.80 to 0.99)	0.024		
Mainly eExercise	2.42	1.99	-17.8	0.83 (0.75 to 0.92)	0.001	0.94 (0.87 to 1.01)	0.108
SHBG‡ (nmol/l)							

Control	42.4	43.2	1.95				
Diet	50.1	58.2	16.1	1.16 (1.08 to 1.24)	<0.001		
Mainly eExercise	50.0	60.4	20.8	1.20 (1.13 to 1.29)	<0.001	1.04 (0.99 to 1.10)	0.128

Women included in this subgroup analysis: *N*=206 (84.7% of total study population). Control, *N*=35 (72.9% of the total control group); Diet, *N*=85 (87.6% of the total diet group); Mainly eExercise, *N*=86 (87.8% of the total mainly exercise group).

Complete case data of oestradiol were available for 198 women; oestrone for 196 women; free oestradiol for 197 women; testosterone and androstenedione for 204 women; free testosterone for 203 women; and SHBG for 205.

‡SHBG: Sex hormone binding globulin

# a *P*-value of <0.025 was considered significant for the comparison of both intervention groups versus control.

^ a *P*-value of <0.05 was considered significant for the comparison mainly exercise versus diet.

*TER=Treatment effect ratio (95% confidence interval), which represents the overall intervention effect on hormone change (adjusted for baseline), estimated by linear regression analysis. Because the linear regression models were based on log-transformed hormone data, the presented treatment effect is the antilogarithm of the original estimate. Therefore, the TER is a ratio that indicates how many times the level in one group is higher (TER>1) or lower (TER<1) compared with a reference group. For example, TER intervention versus control = 0.9 indicates that the hormone level in the intervention group is on average 10% lower compared with the control group.
