# Supplementary material for: Effect of weight loss, with or without exercise, on body composition and sex hormones in postmenopausal women: the SHAPE-2 trial
Source: Breast Cancer Res. 2015 Sep 2;17(1):120. doi: 10.1186/s13058-015-0633-9 (PMC4557857; doi:10.1186/s13058-015-0633-9)
Supplement: Additional file 3: — Effects on sex hormones in women who were adherent to the prescribed physical activity. Secondary analyses of women adherent to the physical activity prescription. (PDF 60.4 kb) [file 13058_2015_633_MOESM3_ESM.pdf]

Additional File 3. Effects on sex hormones in women who were adherent to the prescribed physical activity

	Baseline Geometric mean	16 weeks Geometric mean	% change 16 weeks	TER* (95% CI): Intervention vs. Control	*P*-value#	TER* (95% CI): Mainly e~~E~~xercise vs. Diet	*P*-value^
Estradiol (pg/ml)							
Control	4.49	4.76	6.08				
Diet	4.28	3.60	-15.8	0.78 (0.66 to 0.91)	0.002		
Mainly e~~E~~xercise	3.73	3.19	-14.5	0.75 (0.64 to 0.88)	<0.001	0.96 (0.85 to 1.09)	0.548
Estrone (pg/ml)							
Control	22.9	24.7	7.83				
Diet	20.5	20.1	-1.70	0.88 (0.79 to 0.98)	0.022		
Mainly e~~E~~xercise	19.9	18.5	-7.18	0.82 (0.74 to 0.92)	0.001	0.94 (0.86 to 1.02)	0.134
Free estradiol (pg/ml)							
Control	0.11	0.12	6.54				
Diet	0.10	0.08	-19.1	0.74 (0.62 to 0.87)	<0.001		
Mainly e~~E~~xercise	0.09	0.07	-21.4	0.69 (0.58 to 0.81)	<0.001	0.93 (0.82 to 1.06)	0.294

Testosterone (pg/ml)							
Control	211	208	-1.31				
Diet	200	184	-7.56	0.93 (0.84 to 1.02)	0.132		
Mainly e~~E~~xercise	184	171	-7.01	0.91 (0.83 to 1.01)	0.083	0.99 (0.91 to 1.07)	0.775
Androstenedione (pg/ml)							
Control	654	658	0.53				
Diet	596	536	-10.1	0.86 (0.73 to 1.01)	0.063		
Exercise	563	495	-12.0	0.82 (0.70 to 0.96)	0.016	0.96 (0.85 to 1.08)	0.473
Free testosterone (pg/ml)							
Control	3.05	3.03	-0.65				
Diet	2.64	2.27	-13.8	0.85 (0.76 to 0.95)	0.003		
Mainly e~~E~~xercise	2.40	1.96	-18.3	0.79 (0.71 to 0.88)	<0.001	0.93 (0.86 to 1.02)	0.114
SHBG‡ (nmol/l)							
Control	42.1	41.7	-0.94				
Diet	48.3	53.5	10.7	1.14 (1.05 to 1.24)	0.003		
Mainly e~~E~~xercise	49.8	60.6	21.8	1.26 (1.16 to 1.37)	<0.001	1.10 (1.03 to 1.18)	0.003

Note. Adherence is defined as: for the mainly exercise group, >80% attendance of all exercise classes. For the diet and control group, <60 minutes per week increase in moderate to vigorous activities (≥4MET) based on the SQUASH questionnaire (questions on sports, or transportation, i.e. cycling moderate and high intensity/walking high intensity, or if missing, the Actigraph activity monitor.

Women included in this subgroup analysis: *N*=168 (69.1% of the total study population). Control, n=30 (62.5% of the total control group); Diet, *N*=67 (69.1% of the total diet group); Mainly eExercise, *N*=71 (72.4% of the total mainly exercise group).

Complete case data of oestradiol were available for 164 women; oestrone for 161 women; free oestradiol for 163 women; testosterone and androstenedione for 168 women; free testosterone and SHBG for 167 women.

‡SHBG: Sex hormone binding globulin

# a *P*-value of <0.025 was considered significant for the comparison of both intervention groups versus control.

^ a *P*-value of <0.05 was considered significant for the comparison mainly exercise versus diet.

*TER=Treatment effect ratio (95% confidence interval), which represents the overall intervention effect on hormone change (adjusted for baseline), estimated by linear regression analysis. Because the linear regression models were based on log-transformed hormone data, the presented treatment effect is the antilogarithm of the original estimate. Therefore, the TER is a ratio that indicates how many times the level in one group is higher (TER>1) or lower (TER<1) compared with a reference group. For example, TER intervention versus control = 0.9 indicates that the hormone level in the intervention group is on average 10% lower compared with the control group.
